# Editorial: Insights in biosensors and biomolecular electronics 2024: novel developments, current challenges, and future perspectives

**DOI:** 10.3389/fbioe.2025.1668411

**Published:** 2025-08-28

**Authors:** Guozhen Liu, Zhugen Yang

**Affiliations:** ^1^ Biomedical Engineering Programme, School of Medicine, The Chinese University of Hong Kong, Shenzhen, China; ^2^ Faculty of Engineering and Applied Sciences, Cranfield University, Cranfield, United Kingdom

**Keywords:** biosensors, biomolecular electronics, artificial intelligence, precise diagnosis, challenges

The field of biosensors and biomolecular electronics is at a turning point, with advancements in materials science, nanobiotechnology and engineering approach for device integration, Shi et al. (2024), Yu et al. (2025) and assay development transforming the possibilities for bioanalytical measurement, biomedical applications, and translational research. The Research Topic gathered in this special issue combines fundamental and applied research on real-time neurochemical monitoring, high-throughput cell-based analysis, platform technologies in integrated electronics, and novel protein diagnostic designs. We have outlined the important contributions and assesses their significance of findings for a broad range of application.

*In vivo* monitoring is one of significant challenges associated with biosensors. Park et al. make a significant advance in quantifying neurochemical dynamics by combining an enhanced fast-scan cyclic voltammetry device with a second derivative-based background drift reduction technique. This allows for continuous, long-range measurements of tonic dopamine dynamics in a Parkinson’s disease mice model before and after levodopa administration. The study examines the relationship between the rate of dopamine increase (rather than the cumulative amount) and the progression and severity of levodopa-induced dyskinesia. The research promises a new analytical methodology for drift reduction in electrochemical recordings and applying it to address a relevant pharmacodynamic question in a validated animal model. The findings provide a framework for future *in vivo* behavioral-neurochemical correlation investigations (Park et al.). Lots of other factors such as background signal drifting, *in vivo* stability, device miniaturization, et al. require further study in the future.

Additionally, applying biosensors for cell cytotoxicity is critical in terms of high throughput, endpoint quantification, and reliance on optical detection. Zhao et al. describe the design and deployment of a microwave resonator biosensor using integrated passive device technology to quantify cancer cell growth inhibition in response to chemotherapeutic drugs. The sensor utilizes dielectric permittivity variations induced by variable live cell concentrations to track cytotoxic responses, which are examined here using HepG2 cells and mitomycin C. The assay is verified against the established CCK-8 colorimetric approach, with the resonance amplitude showing a good linear association with live cell quantity and dose-response curves that match those obtained by optical readout. This technology enables non-optical, stain-free, and rapid endpoint measurement with minimal sample volume, achieving a low limit of detection. The technical advancement has major implications for the development of compact, high-throughput, and integratable biosensor systems for cytotoxicity screening and cell analytics (Zhao et al.).

Sensitive, high-throughput protein diagnostics necessitate minimal background and powerful signal amplification, making it difficult to translate modern immunoassay designs into clinical procedures. Hu et al. present a magnetic beads-based proximity extension assay that uses magnetic beads grafted with poly (oligo (ethylene glycol) methacrylate) (POEGMA) brushes. The POEGMA coating possesses antifouling properties, eliminating the need for blocking and lengthy wash steps. Non-specific binding is physically prevented, and capture antibodies are loaded using vacuum-assisted entanglement rather than covalent chemistry. The PEA framework enables dual-antibody identification of a target protein (as demonstrated with IL-8), resulting in PCR-amplifiable DNA only when two oligo-linked antibodies bind the same antigen in proximity. The approach yields limits of detection in the femtogram-per-mL range, which is comparable to digital ELISA, while providing greater assay robustness, decreased procedural complexity, and a workflow that can be completed within an hour. This suggests a possible pathway for clinical translation of multiplexed, high-sensitivity protein panels with scalable throughput (Hu et al.).

Device integration is essential for modern biosensors and molecular electronics. The integration of biosensors with complementary metal-oxide-semiconductor (CMOS) technology to drive the scale, sensitivity, and accessibility of bioelectronic measurement devices was comprehensively reviewed (Dehghandehnavi et al.). Advances on the materials, fabrication techniques, and surface functionalization chemistries that enable cutting-edge CMOS-integrated biosensors for *in vitro* and point-of-care applications are summarized. Meanwhile, authors critically evaluate post-CMOS micro- and nanofabrication processes for producing electrochemically active and biocompatible electrodes, as well as immobilization strategies for functionalizing surfaces with various probes (antibodies, DNA, and aptamers) using covalent, physical, or specific affinity chemistries. The amperometric, potentiometric, impedimetric, and field-effect transistor-based biosensors are technically analyzed, and the development of multimodal, optical, magnetic, and mechanical transduction techniques is included. To address the outstanding challenges, advanced passivation, hydrogels for modular probe coupling, and patternable immobilization are among the proposed solutions, providing a roadmap for next-generation high-performance CMOS biosensors.

The accepted contributions collectively demonstrate the breadth of the biosensors and biomolecular electronics domain. Each article provides robust scientific or engineering solutions to quantitative limits and error causes such as drift, noise, and non-specific binding. From microwave IPD sensors (Sharma and Li, 2025) and monolithic CMOS chips (Wrege et al., 2025) to molecularly designed bead assays (Duffield et al., 2025), systems integration is a clear path toward scalable, multiplexed, and point-of-care diagnostics. Materials engineering, whether functionalizing device surfaces or generating polymer coatings with novel physical-chemical properties, remains critical for improving selectivity, sensitivity, and device robustness. All works address current challenges in biomedical science, such as quantitative neurochemistry, drug screening efficacy, and clinical-grade protein detection, and serve as a bridge between laboratory research and the deployment of future medical technologies.

To fully realize the potential of biosensors and biomolecular electronic systems, future research should focus on scalability in fabrication, reducing user-intensive workflow steps, decoupling device, and assay complexity, and integrating artificial intelligence (AI) (Liu et al., 2024). Meanwhile, the success of biosensors will benefit from the integration with microfluidic devices to realize sample-in-answer-out multiplex detection. AI-driven biosensors and biomolecular electronics rely on data quality, signal analysis algorithms, and sustainable data maintenance. A great emphasis on standardization and clinical validation will be required for widespread clinical adoption, which however are all behind laboratory proof-of-concept. International efforts, such as the FDA Digital Health regulations, National Institutions of Health Rapid Acceleration of Diagnostics, ISO metrology frameworks, et al., are driving the method standardization. Additionally, all biosensing technologies must address questions of cost and environmental impact. CMOS manufacture is energy-intensive yet allows for scale production (possibly lowering per-device costs), whereas microwave and bead-based tests can use small reagent quantities and low-power circuits. Disposability, energy consumption, and toxic byproducts are increasingly scrutinized metrics, particularly for applications in resource-constrained environments. Green chemistry (polymer coatings, biopolymer hydrogels), recyclable device designs, and the reduction of rare elements all deserve more mainstream attention in future research.

On behalf of the editorial team, we would like to extend our appreciation to all authors, reviewers, and contributors for their significant contributions to advancing the scientific excellence and integrity reflected in this Research Topic.
